# Personalized prediction of rehabilitation outcomes in multiple sclerosis: a proof-of-concept using clinical data, digital health metrics, and machine learning

**DOI:** 10.1007/s11517-021-02467-y

**Published:** 2021-11-25

**Authors:** Christoph M. Kanzler, Ilse Lamers, Peter Feys, Roger Gassert, Olivier Lambercy

**Affiliations:** 1 Rehabilitation Engineering Laboratory, Institute of Robotics and Intelligent Systems, Department of Health Sciences and Technology, ETH Zürich, BAA C 307.1, Lengghalde 5, 8008 Zürich, Switzerland; 2 Future Health Technologies, Singapore-ETH Centre, Campus for Research Excellence And Technological Enterprise (CREATE), Singapore, Singapore; 3 REVAL, Rehabilitation Research Center, BIOMED, Biomedical Research Institute, Faculty of Medicine and Life SciencesHasselt University, Hasselt, Belgium; 4 Rehabilitation and MS Center, Pelt, Belgium

**Keywords:** Prognostic factors, Neurorehabilitation, Digital biomarkers, Assessment, Upper limb

## Abstract

Predicting upper limb neurorehabilitation outcomes in persons with multiple sclerosis (pwMS) is essential to optimize therapy allocation. Previous research identified population-level predictors through linear models and clinical data. This work explores the feasibility of predicting individual neurorehabilitation outcomes using machine learning, clinical data, and digital health metrics. Machine learning models were trained on clinical data and digital health metrics recorded pre-intervention in 11 pwMS. The dependent variables indicated whether pwMS considerably improved across the intervention, as defined by the Action Research Arm Test (ARAT), Box and Block Test (BBT), or Nine Hole Peg Test (NHPT). Improvements in ARAT or BBT could be accurately predicted (88% and 83% accuracy) using only patient master data. Improvements in NHPT could be predicted with moderate accuracy (73%) and required knowledge about sensorimotor impairments. Assessing these with digital health metrics over clinical scales increased accuracy by 10%. Non-linear models improved accuracy for the BBT (+ 9%), but not for the ARAT (-1%) and NHPT (-2%). This work demonstrates the feasibility of predicting upper limb neurorehabilitation outcomes in pwMS, which justifies the development of more representative prediction models in the future. Digital health metrics improved the prediction of changes in hand control, thereby underlining their advanced sensitivity.

**Supplementary information:**

The online version contains supplementary material available at 10.1007/s11517-021-02467-y.

**Graphical Abstract:**
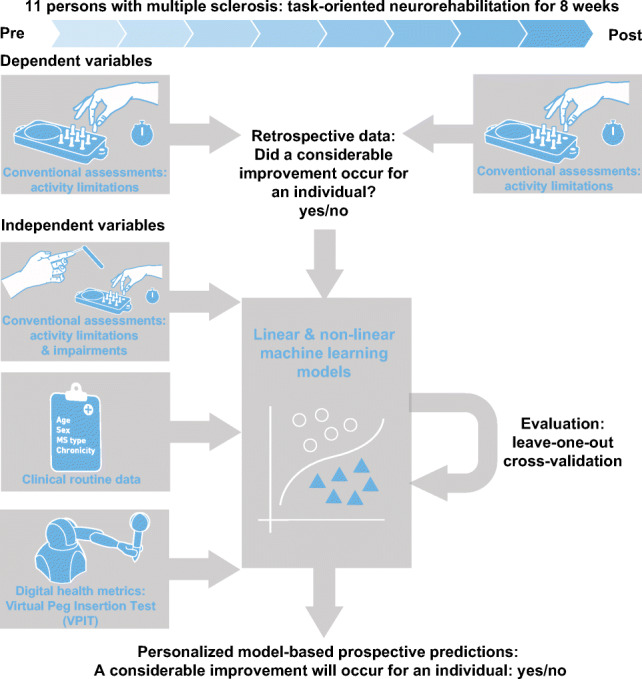
This work explores the feasibility of predicting individual neurorehabilitation outcomes using machine learning, clinical data, and digital health metrics.

## Introduction

Multiple sclerosis (MS) is a chronic neurodegenerative disorder with 2.2 million prevalent cases worldwide [1]. It disrupts a variety of sensorimotor functions and affects the ability to smoothly and precisely articulate complex multi-joint movements, involving, for example, the arm and hand [2]. This strongly affects the ability to perform daily life activities, leads to increased dependence on caregivers, and ultimately reduced quality of life [3]. Inter-disciplinary neurorehabilitation approaches combining, for example, physiotherapy and occupational therapy have shown promise to reduce upper limb disability [4–6]. This is reflected by a reduction in sensorimotor impairments and an increase in the spectrum of executable activities, as defined by the International Classification of Functioning, Disability, and Health (ICF) [7].

One of the active ingredients to ensure successful neurorehabilitation is a careful adaptation of the therapy regimen to the characteristics and deficits of an individual (i.e., personalized therapy) [5, 6, 8]. For this purpose, predicting whether a patient is susceptible to positively respond to a specific neurorehabilitation intervention is of primary interest to researchers and clinicians, as it can help to set more realistic therapy goals, optimize therapy time, and reduce costs related to unsuccessful interventions [9–12]. In addition, it promises to define homogenous and responsive groups for large-scale and resource-intensive clinical trials.

Unfortunately, knowledge about predictors determining the response to neurorehabilitation is limited in pwMS [13–15]. So far, most of the approaches focused on establishing correlations between clinical variables at admission and discharge on a population level. This allowed the identification of, for example, typical routinely collected data (e.g., chronicity) and the severity of initial sensorimotor impairments as factors determining the efficacy of neurorehabilitation [5, 13–15]. However, identifying trends on a population level has limited relevance to actually inform daily clinical decision-making.

Predicting therapy outcomes at an individual level promises to provide more clinically relevant information [9–12], but requires appropriate modeling and evaluation strategies that go beyond the commonly applied linear correlation analyses. More advanced approaches are necessary to account for potentially non-linear relationships and the high behavioral inter-subject variability commonly observed in neurological disorders. In addition, the severity of sensorimotor impairments is often not characterized in a sensitive manner, which might limit their predictive potential. This stems from assessments of sensorimotor impairments that are commonly applied in clinical research (referred to as conventional scales) providing only coarse information, as they usually rely on subjective evaluation or purely timed-based outcomes that are not sufficiently capturing behavioral variability [16, 17].

Machine learning allows accurate and data-driven modeling of complex non-linear relationships, which offers high potential for a precise and personalized prediction of rehabilitation outcomes [18, 19]. Similarly, digital health metrics of sensorimotor impairments allow answering certain limitations of conventional scales by providing objective and fine-grained information without ceiling effects [20]. Such kinematic and kinetic metrics have found first pioneering applications in pwMS, allowing to better disentangle the mechanisms underlying sensorimotor impairments [21–29]. So far, neither of these techniques has been applied for a personalized prediction of rehabilitation outcomes in pwMS.

**Fig. 1:**
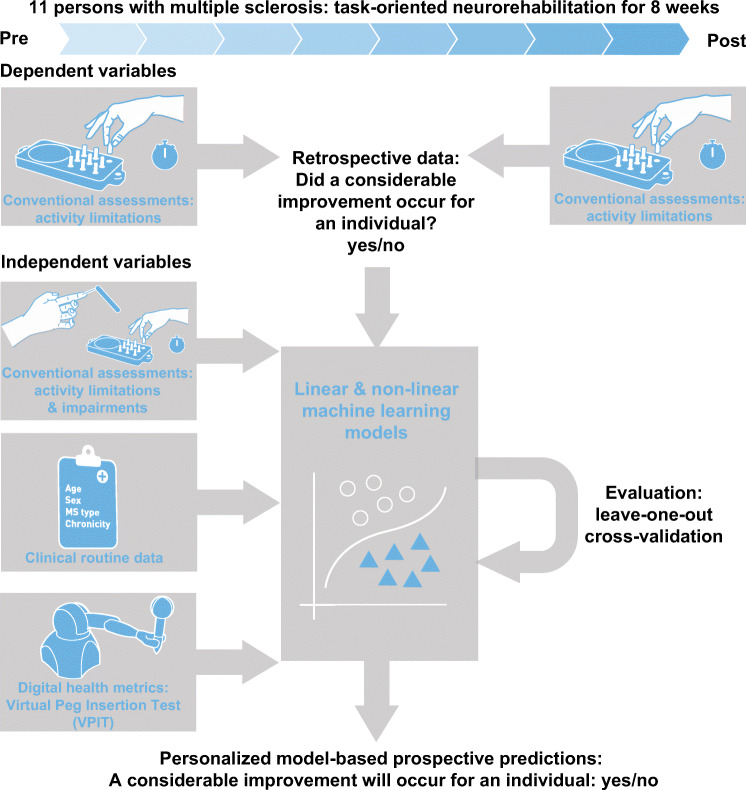
Approach for prediction of neurorehabilitation outcomes in persons with multiple sclerosis. Eleven persons with multiple sclerosis were assessed before and after eight weeks of neurorehabilitation. Multiple linear and non-linear machine learning models were trained on different feature sets with data collected before the intervention. This included information from conventional clinical assessments about activity limitations and impairments, clinical routine data, and digital health metrics collected with the Virtual Peg Insertion Test (VPIT). The dependent variable of the models defined whether a considerable improvement in activity limitations occurred across the intervention or not. The quality and generalizability of the models were evaluated in a leave-one-subject-out cross-validation

The objective of this work was to explore the feasibility of predicting upper limb rehabilitation outcomes in individual pwMS by combining clinical data, digital health metrics, and machine learning (Fig. 1). For this purpose, clinical data including routinely collected information (e.g., age and chronicity) and conventional assessments were recorded pre- and post-intervention in 11 pwMS that participated in a clinical study on task-oriented upper limb rehabilitation [6]. In addition, digital health metrics describing upper limb movement and grip force patterns were recorded using the Virtual Peg Insertion Test (VPIT), a previously validated technology-aided assessment of upper limb sensorimotor impairments relying on a haptic end-effector and a virtual goal-directed object manipulation task [24, 28, 30].

We hypothesized that (1) machine learning models trained on multi-modal data recorded pre-intervention could inform on the possibility to yield a considerable reduction in upper limb disability due to a specific rehabilitation intervention. Further, we assumed that (2) non-linear machine learning models enable more accurate predictions of rehabilitation outcomes than the more commonly applied linear regression approaches. Lastly, we expected (3) digital health metrics of sensorimotor impairments to provide predictive information that goes beyond the knowledge gained from conventional assessments. Successfully addressing these objectives would make an important methodological contribution towards the development of prediction models in pwMS and might allow to speculate about the mechanisms orchestrating sensorimotor recovery. This will pave the way for further research in this area, which could ultimately help optimizing neurorehabilitation planning in pwMS and provide further evidence on its efficacy for healthcare practitioners.

## Methods

### Participants

The data used in this work was collected in the context of a clinical study in which the VPIT was integrated as a secondary outcome measure [6]. The study was a pilot randomized controlled trial on the intensity-dependent effects of technology-aided task-oriented upper limb training at the Rehabilitation and MS centre Pelt (Pelt, Belgium). For this purpose, participants were block randomized based on their disability level into three groups receiving either robot-assisted task-oriented training at 50% or 100% of their maximal possible intensity as defined by their maximum possible number of repetitions of a goal-directed task, or alternatively conventional occupational therapy. The training lasted over a period of 8 weeks with 5h of therapy per week. In addition, participants received standard physical therapy focusing on gait and balance. In total, 11 pwMS that successfully performed the VPIT before the intervention were included in the present work. Other study participants did not complete the VPIT protocol due to severe upper limb disability or strong cognitive deficits. Exclusion criteria and details about the study procedures can be found in previous work describing the clinical outcome of the trial [6]. The study was registered at clinicaltrials.gov (NCT02688231) and approved by the responsible Ethical Committees (University of Leuven, Hasselt University, and Mariaziekenhuis Noord-Limburg).

### Conventional assessments

A battery of established conventional assessments was performed to capture the effects of the interventions. On the ICF body function & structure level, impaired sensation in index finger and thumb were tested using Semmes-Weinstein monofilaments (Smith & Nephew Inc., Germantown, USA) [31]. The results of both tests were combined into a single score (2: normal sensation; 12: maximally impaired sensation). Weakness when performing shoulder abduction, elbow flexion, and pinch grip was rated using the Motricity Index, leading to a single score for all three movements (0: no movement; 100: normal power) [32]. The severity of intention tremor and dysmetria was rated during a finger to nose task using Fahn’s Tremor Rating Scale and summed up to a single score describing tremor intensity (0: no tremor; 6: maximum tremor) [33]. Fatigability was evaluated using the Static Fatigue Index that describes the decline of strength during a 30 s handgrip strength test (0: minimal fatigability; 100: maximal fatigability; [34]). Cognitive impairment was described using the Symbol Digit Modality Test, which defines the number of correct responses in 90 s when learning and recalling the associations between certain symbols and digits [35]. Lastly, the Expanded Disability Status Scale (EDSS; 0: neurologically intact; 10: death) was recorded as an overall disability measure [36].

On the ICF activity level, the Action Research Arm Test (ARAT) evaluated the ability to perform tasks requiring the coordination of arm and hand movements and consists of four parts focusing especially on grasping, gripping, pinching, and gross movements (0: none of the tasks could be completed; 57: all tasks successfully completed without difficulty) [37]. Further, the ability to perform fine dexterous manipulations were described with the time to complete the Nine Hole Peg Test (NHPT) [38, 39]. The capability to execute gross movements was defined through the Box and Block Test (BBT), which defines the number of blocks that can be transferred from one box into another within 1 min [37, 40]. The outcomes of the NHPT and the BBT were defined as *z*-scores based on normative data to account for the influence of sex, age, and the tested body side [40, 41].

### Digital health metrics describing upper limb movement and grip force patterns with the Virtual Peg Insertion Test (VPIT)

The VPIT is a technology-aided assessment that consists of a 3D goal-directed manipulation task (video at https://youtu.be/TyJyd5uVN68). It requires cognitive function as well as the coordination of arm movements, hand movements, and power grip force to insert nine virtual pegs into nine virtual holes [28, 42]. The task is performed with a commercially available haptic end-effector device (PhantomOmni or GeomagicTouch, 3D Systems, USA), a custom-made handle able to record grasping forces, and a virtual reality environment represented on a personal computer. The end-effector device provides haptic feedback that renders the virtual pegboard and its holes. The VPIT protocol consists of an initial familiarization period with standardized instructions followed by five repetitions of the test (i.e., insertion of all nine pegs five times), which should be performed as fast and accurately as possible.

We previously established a processing framework that transforms the recorded kinematic, kinetic, and haptic data into a validated core set of 10 digital health metrics that were objectively selected based on their clinimetric properties [28]. These were defined by considering test-retest reliability, measurement error, and robustness to learning effects (all in neurologically intact participants), the ability of the metrics to accurately discriminate neurologically intact and impaired participants (discriminant validity), as well as the independence of the metrics from each other. The kinematic metrics were extracted during either the transport phase, which is defined as the gross movement from picking up a peg until inserting the peg and requires the application of a grasping force of at least 2 N, or the return phase, which is the gross movement from releasing a peg into a hole until the next peg is picked up and does not require the active control of grasping force. Further, the peg approach and hole approach phases were defined as the precise movements before picking up a peg and releasing it into a hole, respectively.

In the following, the definition and interpretation of the ten core metrics of the VPIT are briefly restated (details in previous and related work [20, 28, 43, 44]). The logarithmic jerk transport, logarithmic jerk return, and spectral arc length return are measures of movement smoothness, which is expected to define the quality of an internal model for movement generation producing appropriately scaled neural commands for the intended movement, and leads to bell-shaped velocity profiles in neurologically intact participants. The jerk-based metrics were calculated by integrating over the third derivative of the position trajectory and by normalizing the outcome with respect to movement length and duration. The spectral arc length was obtained by analyzing the frequency content of the velocity profile. Further, the path length ratio transport and path length ratio return described the efficiency of a movement by comparing the shortest possible distance between start and end of the movement phase and relating this to the actually traveled distance. Additionally, the metric velocity max return describes the maximum speed of the end-effector. The metric jerk peg approach was calculated to capture the behavior during precise movements when approaching the peg. Lastly, three metrics were calculated to capture grip force coordination during transport and hole approach. In more detail, the force rate number of peaks transport (i.e., number of peaks in the force rate profile) and the force rate spectral arc length transport described the smoothness of the force rate signal and were expected to describe abnormal oscillations in the modulation of grip force during gross arm movements. Additionally, the force rate spectral arc length metric was calculated during the hole approach phase.

After the calculation of the metrics, the data processing framework includes the modeling and removal of potential confounds, including age, sex, whether the test was performed with the dominant hand or not, and stereo vision deficits. Lastly, the metrics are normalized with respect to the performance of 120 neurologically intact participants and additionally to the neurologically impaired subject in the VPIT database that showed the worst task performance according to each specific metric. This leads to continuous outcome measures in the unbounded interval $\left ]-\infty \%,+\infty \%\right [$, with the value 0% indicating median task performance of the neurologically intact reference population and 100% the worst task performance of the neurologically affected participants.

### Data analysis

In order to predict neurorehabilitation outcomes, several machine learning models of different complexity were trained on different feature sets (independent variables, details in Table SM3) recorded pre-intervention [45]. Knowledge about whether a participant yielded a considerable reduction of disability on the activity level across the intervention or not was used as the dependent variable for the models (i.e., supervised learning of features with binary ground truth) [45]. A considerable reduction in activity limitations was defined by comparing the change of conventional assessments (ARAT, BBT, or NHPT) to their smallest real difference (SRD) [46]. The SRD defines a range of values for which the assessment cannot distinguish between measurement noise and an actual change in the measured physiological construct. Hence, changes across above the SRD were defined as considerable improvements. Conventional assessments describing activity limitations were used and preferred over a characterization of body functions & structures, as improving the former is more commonly the primary target during neurorehabilitation, and conventional assessments of activity limitations provide more sensitive scales (often continuous time-based) than conventional assessment of body functions (often ordinal) [5]. The SRD for the ARAT, BBT, and NHPT were previously defined for neurological subjects as 5.27 points, 8.11 min^− 1^, and 5.32 s respectively [47–49]. Separate machine learning models were trained for each of these conventional assessments, given that individuals might change only selectively in a subset of these.

The data-driven machine learning models allow generating a transfer function that might be able to associate the value of the independent variables recorded pre-intervention to the rehabilitation outcomes (expected reduction in activity limitations: yes or no). The predictive power of the models was evaluated by comparing the ground truth (i.e., whether a subject significantly reduced its activity limitations across the intervention) with the model estimates for each pwMS via a confusion matrix and the balanced accuracy (i.e., the average of sensitivity and specificity) [50]. This metric was chosen as a primary performance indicator, as it has been recommended as a mathematically robust estimator of a models’ performance and generalizability, even for inbalanced datasets, and allows a concise representation of performance with a single value [50]. For the best performing models, we further reported the sensitivity (true positives over condition positive), specificity (true negatives over condition negative), and precision (true positives over predicted condition positive) to provide a multi-dimensional evaluation of model performance.

As complex machine learning models can theoretically perfectly fit to any type of multi-dimensional data, the models were tested on data that were not used for its training. For this purpose, a leave-one-subject-out cross-validation was applied to train the model on data from all participants except one (i.e., both body sides were removed from the training set). Subsequently, this hold-out dataset was used to test the generalizability of the model. This process was repeated until all possible permutations of testing and training set were covered and is expected to provide a performance evaluation of the model that is more generalizable to unseen data (i.e., assessments on new patients). In addition, the models were specifically evaluated on individuals that have considerable activity limitations pre-intervention but do not show a positive response to neurorehabilitation (i.e., unexpected non-responders), as such patients are of high interest from a clinical perspective. As we hypothesized that different predictive factors influence rehabilitation outcomes, multiple feature sets (i.e., a combination of multiple independent variables) were defined. In more detail, six basic feature sets containing patient master data (MS type, chronicity, age, sex), intervention group, disability level (EDSS and disability information used for block randomization), sensorimotor impairments assessed with conventional scales (motricity index, static fatigue index, monofilament index, symbol digit modality test, Fahn’s tremor rating scale), sensorimotor impairments assessed with the VPIT (ten digital health metrics), and activity limitations assessed with conventional scales (ARAT, BBT, NHPT). Separate machine learning models were trained on these basic feature sets and selected combinations thereof. All feature sets only contained information collected before the intervention.

Four types of machine learning models were used to enable comparisons between linear and non-linear approaches and to ensure the robustness of the results to the model choice. For this purpose, simple models were chosen as these have high interpretability and could be used with standard parameter values to avoid the need for additional parameter validation and to prevent potential overfitting to the dataset. More specifically, decision trees were applied, consisting of multiple nodes that involve the binary testing of a feature based on a threshold, branches that define the outcome of the test (value of feature above or below the threshold), and multiple leaf nodes that indicate a classification label (considerable improvement or not) [51]. The number of nodes, the metric that is tested at each node, and the thresholds are automatically chosen based on a recursive statistical procedure that attempts to minimize the overlap between the distributions of the two classes (considerable improvement or not). This model was chosen due to its simplicity, intuitive interpretability, and high generalizability. In addition, k-nearest neighbor (classification based on normalized Euclidean distances) and random forest (combination of multiple decision trees) models were applied [45]. Finally, a standard linear regression approach was used to establish baseline performance values, given that these are the simplest models and are predominantly used in literature [13–15]. For this approach, the model outputs were rounded to adhere to the binary classification problem. This was preferred over a logistic regression approach, which would be more suitable for binary variables, but would challenge the comparability to existing literature.

## Results

The 11 pwMS (7 female) used for the analysis were of age 56.7 ± 14.8 years and had an EDSS of 6.1 ± 1.3, (mean±standard deviation; detailed information in Table 1). Given that all participants except two successfully completed all assessments with both upper limbs, 20 datasets were available for analysis. Six, nine, and six of these datasets showed considerable improvements in the ARAT, BBT, and NHPT, respectively. One participant (ID 02) was an unexpected non-responder, as he had strong activity limitations (admission: ARAT 44, BBT 20 min^− 1^, NHPT 140.27 s), but did not make considerable improvements during neurorehabilitation (discharge: ARAT 41, BBT 26 min^− 1^, NHPT 216.4 s).

**Table 1:** Clinical information on persons with multiple sclerosis

ID	Time	Side	Age	Sex	MS type	Chronicity	Interv.	EDSS	ARAT	BBT	NHPT
			yrs			yrs		0–10	0–57	1/min	s
01	Pre	Left	52	F	RR	29	1	7	37	22	45.25
01	Post	Left	52	F	RR	29	1	–	49	33	41.14
01	Pre	Right	52	F	RR	29	1	7	47	31	24.75
01	Post	Right	52	F	RR	29	1	–	56	47	23.43
02	Pre	Right	69	M	PP	19	1	7.5	44	20	140.27
02	Post	Right	69	M	PP	19	1	–	41	26	216.4
03	Pre	Left	25	F	RR	6	2	6	52	45	29.35
03	Post	Left	25	F	RR	6	2	–	57	57	23.78
03	Pre	Right	25	F	RR	6	2	6	53	43	29.62
03	Post	Right	25	F	RR	6	2	–	55	52	23.81
04	Pre	Left	42	F	RR	1	1	4	56	39	27.81
04	Post	Left	42	F	RR	1	1	–	56	57	23.52
04	Pre	Right	42	F	RR	1	1	4	54	40	20.48
04	Post	Right	42	F	RR	1	1	–	55	65	20.92
05	Pre	Left	56	F	SP	10	2	7	49	38	33.72
05	Post	Left	56	F	SP	10	2	–	54	44	35.3
05	Pre	Right	56	F	SP	10	2	7	29	25	89.79
05	Post	Right	56	F	SP	10	2	–	40	28	70.24
06	Pre	Left	65	M	SP	19	2	8	52	34	39.9
06	Post	Left	65	M	SP	19	2	–	54	34	44.13
07	Pre	Left	63	F	RR	8	2	4.5	57	60	20.84
07	Post	Left	63	F	RR	8	2	–	57	49	22.28
07	Pre	Right	63	F	RR	8	2	4.5	54	47	35.04
07	Post	Right	63	F	RR	8	2	–	55	45	27.3
08	Pre	Left	76	F	RR	38	1	5	43	42	27.01
08	Post	Left	76	F	RR	38	1	–	54	56	24.61
08	Pre	Right	76	F	RR	38	1	5	34	43	34.46
08	Post	Right	76	F	RR	38	1	–	54	49	23.46
09	Pre	Left	60	M	PP	21	1	7	52	44	31.48
09	Post	Left	60	M	PP	21	1	–	54	49	35.66
09	Pre	Right	60	M	PP	21	1	7	53	51	25.29
09	Post	Right	60	M	PP	21	1	–	55	48	41.93
10	Pre	Left	46	M	PP	11	2	5.5	55	32	30.58
10	Post	Left	46	M	PP	11	2	–	56	43	39.08
10	Pre	Right	46	M	PP	11	2	5.5	56	35	23.23
10	Post	Right	46	M	PP	11	2	–	56	53	20.83
11	Pre	Left	70	F	RR	37	3	6	53	45	29.86
11	Post	Left	70	F	RR	37	3	–	55	39	35.28
11	Pre	Right	70	F	RR	37	3	6	45	42	53.21
11	Post	Right	70	F	RR	37	3	–	52	41	46.19

Subject 2 was defined as a unexpected non-responder, as he had the strongest activity limitations at admission, but did not respond positively to neurorehabilitation. ID: participant identifier. F: female. M: male. Intervention (interv.) group: task-oriented high intensity (1), task-oriented low intensity (2), control (3). RR: relapse remitting. PP: primary progressive. SP: secondary progressive. EDSS: Expanded Disability Status Scale. NHPT: Nine Hole Peg Test. BBT: Box and Block Test. ARAT: Action Research Arm Test. VPIT: Virtual Peg Insertion Test

The performance evaluation for machine learning models trained on different feature sets and different conventional scores can be found in Table 2 (k-nearest neighbor), Table SM4 (linear regression), Table SM5 (decision tree), and Table SM6 (random forest). Table 3 provides a detailed evaluation of the best performing models.

**Table 2:** Predicting intervention outcomes using data collected pre-intervention and a k-nearest neighbor model

	Machine learning: k-nearest neighbor model					
Feature sets	All participants			Unexpected non-responder		
	Outcome prediction for			Outcome prediction for		
	ARAT	BBT	NHPT	ARAT	BBT	NHPT
	Balanced accuracy (%)			Correct (yes/no)		
1	55	63	43	y	y	y
2	49	28	54	n	n	y
3	52	37	43	y	y	y
4	43	53	40	n	n	y
5	77	66	**71**	y	y	y
6	80	63	64	n	y	n
1, 2	**83**	**80**	43	y	y	y
1, 3	60	25	50	y	y	y
1, 4	55	46	63	y	y	y
1, 5	69	55	64	y	y	y
1, 6	93	59	33	n	y	n
1, 2, 3	71	35	50	y	y	y
1, 4, 6	68	42	48	n	y	n
1, 5, 6	85	60	68	n	y	y
1, 4, 5, 6	64	69	61	n	y	n
1, 2, 3, 4, 5, 6	68	59	64	n	y	y

Multiple machine learning models were trained using different feature sets (independent variables, 1–6). The training label indicated whether a considerable change across intervention was observed in a specific conventional score (dependent variable; ARAT, BBT, or NHPT). The models were evaluated in a leave-one-out cross-validation and specifically tested for one individual with strong activity limitations who did not show improvements across neurorehabilitation (referred to as unexpected non-responder). Feature set nomenclature: (1) patient master data (ms type, chronicity, age, sex); (2) intervention group; (3) disability (EDSS, disability group); (4) conventional scales of body functions (motricity index, static fatigue index, monofilament index, symbol digit modality test, Fahn’s tremor rating scale); (5) digital health metrics of sensorimotor impairments (ten VPIT metrics); (6) Conv. scale of activity (ARAT, NHPT, BBT). The best performing (accuracy and unexpected non-responder) models relying on the least amount of features are highlighted in bold for each conventional scale. ARAT: Action Research Arm Test. BBT: Box and Block Test. NHPT: Nine Hole Peg Test. VPIT: Virtual Peg Insertion Test

**Table 3:** Predicting intervention outcomes in ARAT, BBT, and NHPT using data collected pre-intervention — detailed performance of best performing models

	Best performing models and feature sets					
	Model	Feature set	Balanced accuracy (%)	Sensitivity (%)	Specificity (%)	Precision (%)
ARAT	Linear regression	1, 6	89	100	79	67
BBT	Decision tree	1	83	67	100	100
NHPT	Linear regression	5	73	67	79	57

Best performing models were selected according to the balanced accuracy and their ability to correctly identify the unexpected non-responder. Feature set nomenclature: (1) patient master data (ms type, chronicity, age, sex); (2) intervention group; (3) disability (EDSS, disability group); (4) conventional scales of body functions (motricity index, static fatigue index, monofilament index, symbol digit modality test, Fahn’s tremor rating scale); (5) digital health metrics of sensorimotor impairments (ten VPIT metrics); (6) conventional scale of activity (ARAT, NHPT, BBT)

The decision tree models that performed best (i.e., models with maximum balanced accuracy that also correctly predicted the unexpected non-responder) predicted changes in ARAT and BBT with a cross-validated balanced accuracy of 88% and 83%, respectively, and relied only on patient master data. The best decision tree predicting changes in NHPT relied purely on digital health metrics of sensorimotor impairments, yielding a balanced accuracy of 49%. The best linear regression model achieved a balanced accuracy of 89% for the ARAT (independent variables: patient master data and conventional scales of activity), 74% for the BBT (patient master data and intervention group), and 73% for the NHPT (digital health metrics). The best k-nearest neighbor models achieved a balanced accuracy of 83% for the ARAT (independent variables: patient master data and intervention type), 80% for the BBT (patient master data and intervention type), and 71% for the NHPT (digital health metrics). The best random forest models achieved a balanced accuracy of 71% for the ARAT (independent variables: patient master data), 83% for the BBT (patient master data), and 67% for the NHPT (patient master data, conventional scales of body function and activity, digital health metrics).

Non-linear machine learning models had similar predictive or did slightly improve predictive performance compared to linear models (ARAT -1%, BBT + 9%, NHPT -2%). For models predicting changes in NHPT that relied solely on conventional scales of body function or digital health metrics, the ones relying on digital health metrics improved predictive accuracy by + 11% for decision tree, + 24% for linear regression, + 31% for k-nearest neighbor, and -8% for random forest models.

The best performing model (balanced accuracy and non-responder) for predicting changes in the NHPT that relied on conventional scales of body functions but not digital health metrics achieved an accuracy of 63% (k-nearest neighbor, patient meta data and conventional scales of body function). The best performing model for predicting changes in the NHPT that relied on digital health metrics but not on conventional scales of body functions achieved an accuracy of 73% (linear regression, digital health metrics).

## Discussion

The objective of this work was to explore the feasibility of predicting the response of individual pwMS to specific upper limb neurorehabilitation interventions by applying machine learning to clinical data and digital health metrics recorded pre-intervention. For this purpose, patient master data, conventional scales describing body functions and activities, as well as upper limb movement and grip force patterns were recorded in 11 pwMS that received eight weeks of neurorehabilitation. Four commonly applied machine learning models (decision tree, random forest, k-nearest neighbor, linear regression) were trained on six different feature sets and combinations thereof. The models were evaluated based on their ability to correctly predict the presence of changes in activity limitations across the intervention and based on their ability to accurately anticipate outcomes for one subject with strong activity limitations at admission but without significant gains across intervention (i.e., an unexpected non-responder).

In summary, changes in ARAT or BBT could be accurately predicted (88% and 83% balanced accuracy, respectively) by only relying on patient master data (namely age, sex, MS type and chronicity). Moreover, changes in NHPT could be predicted with moderate accuracy (73% balanced accuracy), but only when providing the models with information about sensorimotor impairments. Assessing these with digital health metrics as provided by the VPIT improved predictive performance by + 10% compared to conventional assessments.

### Machine learning enables a personalized prediction of rehabilitation outcomes in pwMS

These results successfully demonstrate the feasibility of predicting the response of individual pwMS to specific neurorehabilitation interventions using machine learning and multi-modal clinical and behavioral data. This work especially makes an important methodological contribution, as it is the first attempt towards a personalized prediction of neurorehabilitation outcomes in pwMS. So far, such approaches were rather employed to predict natural disease progression in pwMS [52–57]. Previous work in neurorehabilitation of pwMS focused on predicting adherence to telerehabilitation [58] or identifying population-level predictors of therapy outcomes through linear regression [13–15]. For the latter, the models were commonly evaluated by comparing the amount of variance explained by the model with the overall variance, which only provides population-level information and challenges comparisons across models trained on different dependent variables [59, 60]. The presented methodology expands this work by applying an in-depth evaluation with accepted performance metrics (primary: balanced accuracy; secondary: sensitivity, specificity, and precision) that can be directly related to the predictive performance for an individual patient and, thus, have higher clinical relevance. The high sensitivity (100%) but moderate precision (67%) for the ARAT models suggests that they tend to overestimate the recovery potential of a patient. For the BBT models, the opposing behavior was observed (67% sensitivity, 100% precision), suggesting too conservative predictions. For the NHPT, specificity was moderate (79%), but sensitivity (67%) and precision (57%) remained low. This highlights that changes in fine hand control are most challenging to predict and underlines the potential for follow-up studies with more representative datasets to further optimize predictive performance.

Further, the non-linear machine learning models applied in this work were able to selectively improve (+ 9% for the BBT) the predictive accuracy compared to linear regression approaches. This suggests the relevance of advanced modeling techniques to explore potential non-linearities between predictors and rehabilitation outcomes.

The four machine learning models were selected based on their robustness, as they are known to perform well on rather small datasets [45]. In addition, these models do not require the optimization of specific input parameters or model architectures, as compared to, for example, more complex neural network-based models [45, 61]. Hence, this promises that other researchers can easily adopt the presented methodology. In addition, it should be emphasized that all models were generated in a data-driven manner. In the presented context, such data-driven approaches are preferable over models that require the manual definition of a mathematical formula (e.g., non-linear mixed effect models), which can introduce bias and require advanced knowledge about expected patterns of recovery that is unfortunately often not available.

### Clinical applicability and mechanisms underlying the prediction of neurorehabilitation outcomes

Patient master data was sufficient to accurately predict changes in the ARAT and BBT. Given that this information is typically available for every patient undergoing neurorehabilitation, such a model could be easily integrated into daily clinical decision-making. Therein, the objective output of the model could complement other, often more subjective, information that is used by healthcare practitioners to define patient-specific therapy programs and set rehabilitation goals [4, 5]. As the proposed models seem to be able to identify non-responders to a restorative neurorehabilitation intervention strategy, this could allow to rather focus, for such individuals, on approaches aiming at learning compensatory strategies in order to improve their spectrum of activities, their quality of life and participation in the community. On the other hand, individuals identified as responders might instead benefit from therapy aimed at the neuroplastic restoration of impaired body functions [11, 62]. While the specific mechanisms underlying the predictive power of patient master data remain unclear, we speculate that these data affect multiple aspects that determine the success of neurorehabilitation, for example, the biological substrates for neuroplasticity, participation in therapy, and learned non-use [11, 58, 62, 63]. The latter might play an especially important role, given that individuals with higher chronicity showed significantly larger gains in the ARAT (*r* = 0.58, *p* < 0.001, Figure SM1). Surprisingly, younger pwMS showed significantly larger gains in the BBT (*r* = − 0.52, *p* < 0.001, Figure SM1), whereas both age and chronicity did not have a significant effect on changes in the NHPT. In the future, this selective and partially opposing effect of age and chronicity needs to be fully elucidated in adequate samples. Also, knowledge of the intervention type (i.e., task-oriented therapy at high or moderate intensity, or occupational therapy) did not considerably improve predictive performance. While stronger intensity-dependent effects were found in the clinical analysis of this trial [6], its rather minor impact in the analysis presented here might be explained by the reduced number of datasets being available for this work, with only two of them belonging to the occupational therapy group.

Interestingly, information about sensorimotor impairments was necessary to predict changes in the NHPT. This indicates that, most likely, different mechanisms underlie the observed improvements in ARAT and BBT scores compared to NHPT outcomes. While more advanced analysis and large-scale studies would be necessary to fully unravel these mechanisms, we speculate that changes in the ARAT and BBT are influenced by multiple factors such as hand control, voluntary neural drive, weakness, fatigue, and attentive deficits, whereas changes in the NHPT might more reflect the recovery of sensorimotor function needed to perform fine dexterous finger movements. One could carefully speculate that this dexterous hand function might be linked to the integrity of the corticospinal tract, which has been shown to be essential for sensorimotor recovery in other neurological disorders [64, 65] and might also play a role in pwMS [66].

### Digital health metrics outperformed conventional scales for predicting changes in the NHPT

The digital health metrics of sensorimotor impairments extracted from the VPIT outperformed conventional scales of sensorimotor impairments when predicting changes in activity limitations, as measured by the NHPT. Hence, we argue that the proposed digital health metrics allow, for this specific application, a superior evaluation of sensorimotor impairments than conventional scales. This is likely because the former provide continuous fine-graded information on ratio scales that might be beneficial for training the machine learning models, compared to the more coarse ordinal scales of conventional assessments. Also, the superiority of digital health metrics for predicting rehabilitation outcomes might be explained by none of the conventional assessments being able to provide metrics specifically capturing impaired grip force coordination as done by the VPIT.

When comparing the VPIT to other technology-aided assessments in pwMS, it becomes apparent that most of them focus more on the evaluation of arm movements with less focus on the hand [21–23, 25–27, 29], which seems to be especially important for relating impairments to their functional impact. Overall, the VPIT emerges as a unique tool able to provide digital health metrics, which complement the clinically available information about impaired body functions. In addition, the assessments with the VPIT can be performed within approximately 15 min per upper limb, thereby showing high clinical feasibility.

### Limitations

A major limitation of this work is the small sample size included for the training and evaluation of the machine learning models. In addition, given the slight imbalance between numbers of pwMS with and without considerable changes in activity limitations across the intervention, it might be that the models slightly overfitted to the group with more observations. Hence, it is unlikely that the current models would accurately generalize to the heterogeneous population of all pwMS. Further, it is unclear whether the models would be able to predict the effect of a different type of neurorehabilitation intervention or whether therapy parameters would need to be integrated into the model. As any related study, this work is also limited by the specific conventional scales and digital health metrics that were used to quantify impaired body functions and activity limitations. Therefore, it is unclear whether different trends would be observed when considering other conventional or instrumented assessments. Lastly, the predictive performance of the models needs to be further optimized, especially with a focus on the precision of the ARAT and NHPT predictions (Table 3).

## Conclusions

This work successfully established the feasibility of an individualized prediction of upper limb neurorehabilitation outcomes in pwMS by combining machine learning with multi-modal clinical and behavioral data collected before a neurorehabilitation intervention. Information about sensorimotor impairments was necessary to predict changes in fine dexterous hand control. In these cases, conventional scales of impaired body functions were outperformed in terms of predictive power by digital health metrics, thereby underlining their potential to provide a more sensitive and fine-grained assessment. Ultimately, this work has the potential to inform future research in the prediction of neurorehabilitation outcomes in pwMS and other neurological conditions.

Future work should focus on validating these results in large-scale populations in order to build models that are more representative of the heterogeneous population of pwMS and can be seamlessly integrated into daily clinical routine. These models should include more holistic information on each individual, including for example information about their psychological status and intrinsic motivation, thereby promising higher prediction accuracies. Also, pivoting from the proposed classification (binary output) towards a regression (continuous output) approach will allow providing a higher level of granularity in the predicted outcomes. Lastly, the inclusion of additional therapy parameters in the models could enable in silico clinical trials, thereby allowing to predict the effects of different therapies for each individual and support a more optimal and data-driven clinical decision-making process.

## Electronic supplementary material

Below is the link to the electronic supplementary material.

(PDF 151 KB )

## Abbreviations

ARAT: Action Research Arm Test
BBT: Box and Block Test
EDSS: Expanded Disability Status Scale
ICF: International Classification of Functioning, Disability, and Health
MS: Multiple Sclerosis
NHPT: Nine Hole Peg Test
pwMS: Persons with Multiple Sclerosis
SRD: Smallest Real Difference
VPIT: Virtual Peg Insertion Test

## References

[1] Wallin MT, Culpepper WJ, Nichols E, Bhutta ZA, Gebrehiwot TT, Hay SI, Khalil IA, Krohn KJ, Liang X, Naghavi M, Mokdad AH, Nixon MR, Reiner RC, Sartorius B, Smith M, Topor-Madry R, Werdecker A, Vos T, Feigin VL, Murray CJ (2019). Global, regional, and national burden of multiple sclerosis 1990–2016: a systematic analysis for the Global Burden of Disease Study 2016. Lancet Neurol.

[2] Browne P, Chandraratna D, Angood C, Tremlett H, Baker C, Taylor BV, Thompson AJ (2014). Atlas of Multiple Sclerosis 2013: A growing global problem with widespread inequity. Neurology.

[3] Yozbatiran N, Baskurt F, Baskurt Z, Ozakbas S, Idiman E (2006). Motor assessment of upper extremity function and its relation with fatigue, cognitive function and quality of life in multiple sclerosis patients. J Neurol Sci.

[4] Khan F, Turner-Stokes L, Ng L, Kilpatrick T, Amatya B (2007) Multidisciplinary rehabilitation for adults with multiple sclerosis. Cochrane Database Syst Rev (2) 10.1002/14651858.CD006036.pub2 PMC8992048 17443610

[5] Beer S, Khan F, Kesselring J (2012). Rehabilitation interventions in multiple sclerosis: An overview. J Neurol.

[6] Lamers I, Raats J, Spaas J, Meuleman M, Kerkhofs L, Schouteden S, Feys P (2019). Intensity-dependent clinical effects of an individualized technology-supported task-oriented upper limb training program in Multiple sclerosis: A pilot randomized controlled trial. Mult Scler Relat Disord.

[7] World Health Organization (2001) International classification of functioning, disability and health: ICF

[8] Khan F, Amatya B (2017). Rehabilitation in multiple sclerosis: a systematic review of systematic reviews. Arch Phys Med Rehabil.

[9] Reinkensmeyer DJ, Burdet E, Casadio M, Krakauer JW, Kwakkel G, Lang CE, Swinnen SP, Ward NS, Schweighofer N (2016). Computational neurorehabilitation: modeling plasticity and learning to predict recovery. J NeuroEng Rehab.

[10] Stinear C (2010). Prediction of recovery of motor function after stroke. Lancet Neurol.

[11] Lipp I, Tomassini V (2015). Neuroplasticity and motor rehabilitation in multiple Sclerosis. Front Neurol.

[12] Stinear CM, Smith MC, Byblow WD (2019). Prediction tools for stroke rehabilitation. Stroke.

[13] Heinemann AW, Linacre JM, Wright BD, Hamilton BB, Granger C (1994). Prediction of rehabilitation outcomes with disability measures. Arch Phys Med Rehabil.

[14] Langdon DW, Thompson AJ (1999). Multiple sclerosis: A preliminary study of selected variables affecting rehabilitation outcome. Mult Scler.

[15] Grasso MG, Troisi E, Rizzi F, Morelli D, Paolucci S (2005). Prognostic factors in multidisciplinary rehabilitation treatment in multiple sclerosis: An outcome study. Mult Scler.

[16] Lamers I, Kelchtermans S, Baert I, Feys P (2014). Upper limb assessment in multiple sclerosis: A systematic review of outcome measures and their psychometric properties. Arch Phys Med Rehabil.

[17] Burridge J, Alt Murphy M, Buurke J, Feys P, Keller T, Klamroth-Marganska V, Lamers I, McNicholas L, Prange G, Tarkka I, Timmermans A, Hughes A-M (2019). A systematic review of international clinical guidelines for rehabilitation of people with neurological conditions: what recommendations are made for upper limb assessment?. Front Neurol.

[18] Obermeyer Z, Emanuel EJ (2016). Predicting the future — big data, machine learning, and clinical medicine. New England J Med.

[19] Rajkomar A, Dean J, Kohane I (2019). Machine learning in medicine. N Engl J Med.

[20] Schwarz A, Kanzler CM, Lambercy O, Luft AR, Veerbeek JM (2019). Systematic review on kinematic assessments of upper limb movements after stroke. Stroke.

[21] Bardorfer A, Munih M, Zupan A, Primožič A (2001). Upper limb motion analysis using haptic interface. IEEE/ASME Trans Mechatron.

[22] Vergaro E, Squeri V, Brichetto G, Casadio M, Morasso P, Solaro C, Sanguineti V (2010). Adaptive robot training for the treatment of incoordination in Multiple Sclerosis. J NeuroEng Rehab.

[23] Carpinella I, Cattaneo D, Bertoni R, Ferrarin M (2012). Robot training of upper limb in multiple sclerosis: comparing protocols with or withoutmanipulative task components. IEEE Trans Neural Sys Rehab Eng.

[24] Lambercy O, Fluet M-C, Lamers I, Kerkhofs L, Feys P, Gassert R (2013) Assessment of upper limb motor function in patients with multiple sclerosis using the virtual peg insertion test: A pilot study. In: Proceedings of the international conference on rehabilitation robotics (ICORR), pp 1–6 10.1109/ICORR.2013.6650494 24187309

[25] Maris A, Coninx K, Seelen H, Truyens V, De Weyer T, Geers R, Lemmens M, Coolen J, Stupar S, Lamers I, Feys P (2018). The impact of robot-mediated adaptive I-TRAVLE training on impaired upper limb function in chronic stroke and multiple sclerosis. Disab Rehab Assist Technol.

[26] Carpinella I, Cattaneo D, Ferrarin M (2014). Quantitative assessment of upper limb motor function in multiple sclerosis using an instrumented action research arm test. J NeuroEng Rehab.

[27] Pellegrino L, Coscia M, Muller M, Solaro C, Casadio M (2018). Evaluating upper limb impairments in multiple sclerosis by exposure to different mechanical environments. Scient Rep.

[28] Kanzler CM, Rinderknecht MD, Schwarz A, Lamers I, Gagnon C, Held J, Feys P, Luft AR, Gassert R, Lambercy O (2020). A data-driven framework for the selection and validation of digital health metrics:, use-case in neurological sensorimotor impairments. npj Digit Med.

[29] Simmatis LE, Jin AY, Taylor SW, Bisson EJ, Scott SH, Baharnoori M (2020) The feasibility of assessing cognitive and motor function in multiple sclerosis patients using robotics. Mult Scler J Exper Transl Clin 6(4) 10.1177/2055217320964940 PMC7580159 33149931

[30] Kanzler CM, Schwarz A, Held J, Luft AR, Gassert R, Lambercy O (2020). Technology-aided assessment of functionally relevant sensorimotor impairments in arm and hand of post-stroke individuals. J NeuroEng Rehab.

[31] Bell-Krotoski J, Tomancik E (1987). The repeatability of testing with Semmes-Weinstein monofilaments. J Hand Surg.

[32] Demeurisse G, Demol O, Robaye E (1980). Motor evaluation in vascular hemiplegia. Eur Neurol.

[33] Fahn S, Tolosa E, Marín C (1993). Clinical rating scale for tremor. Park Dis Mov Disord.

[34] Surakka J, Romberg A, Ruutiainen J, Aunola S, Virtanen A, Karppi S-L, Mäentaka K (2004). Effects of aerobic and strength exercise on motor fatigue in men and women with multiple sclerosis: a randomized controlled trial. Clin Rehab.

[35] Benedict RH, DeLuca J, Phillips G, LaRocca N, Hudson LD, Rudick R (2017). Validity of the symbol digit modalities test as a cognition performance outcome measure for multiple sclerosis. Mult Scler J.

[36] Kurtzke JF (1983). Rating neurologic impairment in multiple sclerosis: an expanded disability status scale (edss). Neurology.

[37] Platz T, Pinkowski C, van Wijck F, Kim I-H, di Bella P, Johnson G (2005). Reliability and validity of arm function assessment with standardized guidelines for the fugl-meyer test, action research arm test and box and block test: a multicentre study. Clin Rehab.

[38] Mathiowetz V, Weber K, Kashman N, Volland G (1985). Adult norms for the nine hole peg test of finger dexterity. Occupat Therapy J Res.

[39] Feys P, Lamers I, Francis G, Benedict R, Phillips G, Larocca N, Hudson LD, Rudick R (2017). The nine-hole peg test as a manual dexterity performance measure for multiple sclerosis. Mult Scler J.

[40] Mathiowetz V, Volland G, Kashman N, Weber K (1985). Adult norms for the box and block test of manual dexterity. Amer J Occupat Therapy.

[41] Mitchell A, Le V, Muniz S, Vogel KA, Vollmer MA, Oxford Grice K (2010) Adult norms for a commercially available nine hole peg test for finger dexterity. Am J Occup Ther 57(5):570–573 10.5014/ajot.57.5.570 14527120

[42] Fluet M, Lambercy O, Gassert R (2011) Upper limb assessment using a virtual peg insertion test. In: IEEE international conference on rehabilitation robotics, pp 1–6 10.1109/ICORR.2011.5975348 22275552

[43] Hogan N, Sternad D (2009). Sensitivity of smoothness measures to movement duration, amplitude, and arrests. J Motor Behav.

[44] Balasubramanian S, Melendez-Calderon A, Roby-Brami A, Burdet E (2015). On the analysis of movement smoothness. J Neuroeng Rehab.

[45] Hastie T, Tibshirani R, Friedman J (2009). The elements of statistical learning, ser. Springer Series in Statistics, no. 1.

[46] Schuck P, Zwingmann C (2003). The ’smallest real difference’ as a measure of sensitivity to change: A critical analysis. Int J Rehabil Res.

[47] De Groot V, Beckerman H, Uitdehaag BM, De Vet HC, Lankhorst GJ, Polman CH, Bouter LM (2006). The usefulness of evaluative outcome measures in patients with multiple sclerosis. Brain.

[48] Paltamaa J, Sarasoja T, Leskinen E, Wikstrom J, Malkia E (2008). Measuring deterioration in international classification of functioning domains of people with multiple sclerosis who are ambulatory. Phys Therapy.

[49] Fritz SL, Blanton S, Uswatte G, Taub E, Wolf SL (2009). Minimal detectable change scores for the wolf motor function test. Neurorehab Neural Repair.

[50] Brodersen KH, Ong CS, Stephan KE, Buhmann JM (2010) The balanced accuracy and its posterior distribution. Proc Int Conf Patt Recogn: 3121–3124

[51] Breiman L (2017) Classification and regression trees

[52] Runmarker B, Andersson C, Odén A, Andersen O (1994). Prediction of outcome in multiple sclerosis based on multivariate models. J Neurol.

[53] Fiorini S, Verri A, Barla A, Tacchino A, Brichetto G (2017) Temporal prediction of multiple sclerosis evolution from patient-centered outcomes. In: Proceedings of the 2nd Machine Learning for Healthcare Conference, ser. Proceedings of Machine Learning Research, vol 68, pp 112–125

[54] Castle D, Wynford-Thomas R, Loveless S, Bentley E, Howell OW, Tallantyre EC (2019). Using biomarkers to predict clinical outcomes in multiple sclerosis. Pract Neurol.

[55] Law MT, Traboulsee AL, Li DK, Carruthers RL, Freedman MS, Kolind SH, Tam R (2019) Machine learning in secondary progressive multiple sclerosis: an improved predictive model for short-term disability progression. Mult Scler J Exper Translat Clin 5(4) 10.1177/2055217319885983 PMC6836306 31723436

[56] Tousignant A, Paul Lemaitre M, Doina Precup C, Arnold DL (2019). Prediction of disease progression in multiple sclerosis patients using deep learning analysis of MRI data tal arbel 3. Proc Machine Learn Res.

[57] Brichetto G, Monti Bragadin M, Fiorini S, Battaglia MA, Konrad G, Ponzio M, Pedullà L, Verri A, Barla A, Tacchino A (2020). The hidden information in patient-reported outcomes and clinician-assessed outcomes: multiple sclerosis as a proof of concept of a machine learning approach. Neurol Sci.

[58] Jeong IC, Liu J, Finkelstein J (2019). Factors affecting adherence with telerehabilitation in patients with multiple sclerosis. Stud Health Technol Inform.

[59] Hamilton DF, Ghert M, Simpson AHRW (2015). Interpreting regression models in clinical outcome studies. Bone Joint Res.

[60] Roy K, Das RN, Ambure P, Aher RB (2016). Be aware of error measures. Further studies on validation of predictive QSAR models. Chemometr Intell Lab Syst.

[61] Goodfellow I, Bengio Y, Courville A (2016) Deep learning

[62] Tomassini V, Matthews PM, Thompson AJ, Fuglø D, Geurts JJ, Johansen-Berg H, Jones DK, Rocca MA, Wise RG, Barkhof F, Palace J (2012). Neuroplasticity and functional recovery in multiple sclerosis. Nat Rev Neurol.

[63] Barghi A, Allendorfer JB, Taub E, Womble B, Hicks JM, Uswatte G, Szaflarski JP, Mark VW (2018). Phase II randomized controlled trial of Constraint-Induced movement therapy in multiple sclerosis. Part 2: effect on white matter integrity. Neurorehabil Neural Repair.

[64] Stinear CM, Barber PA, Smale PR, Coxon JP, Fleming MK, Byblow WD (2007). Functional potential in chronic stroke patients depends on corticospinal tract integrity. Brain.

[65] Stinear CM, Barber PA, Petoe M, Anwar S, Byblow WD (2012). The PREP algorithm predicts potential for upper limb recovery after stroke. Brain.

[66] Neva JL, Lakhani B, Brown KE, Wadden KP, Mang CS, Ledwell NH, Borich MR, Vavasour IM, Laule C, Traboulsee AL, MacKay AL, Boyd LA (2016). Multiple measures of corticospinal excitability are associated with clinical features of multiple sclerosis. Behav Brain Res.

