# Supplementary material for: Personalized prediction of rehabilitation outcomes in multiple sclerosis: a proof-of-concept using clinical data, digital health metrics, and machine learning
Source: Med Biol Eng Comput. 2021 Nov 25;60(1):249–61. doi: 10.1007/s11517-021-02467-y (PMC8724183; doi:10.1007/s11517-021-02467-y)
Supplement: Supplementary file 1 — (PDF 151 KB ) [file 11517_2021_2467_MOESM1_ESM.pdf]

Supplementary material

Personalized prediction of rehabilitation outcomes in multiple sclerosis: a proof-of-concept using clinical data, digital health metrics, and machine learning

Christoph M. Kanzler, PhD[1,2], Ilse Lamers, PhD[3,4], Peter Feys, PhD[3], Roger Gassert, PhD[1], and Olivier Lambercy, PhD[1]

Medical & Biological Engineering & Computing
06/2021

1 Rehabilitation Engineering Laboratory, Institute of Robotics and Intelligent Systems, Department of Health Sciences and Technology, ETH Zürich, Switzerland.
2 Future Health Technologies, Singapore-ETH Centre, Campus for Research Excellence And Technological Enterprise (CREATE), Singapore.
3 REVAL, Rehabilitation Research Center, BIOMED, Biomedical Research Institute, Faculty of Medicine and Life Sciences, Hasselt University, Belgium.
4 Rehabilitation and MS center, Pelt, Belgium.

Corresponding author: Christoph M. Kanzler, Rehabilitation Engineering Laboratory, ETH Zürich, BAA C 307.1, Lengghalde 5, 8008 Zürich, Switzerland. Email: christoph.kanzler@sec.ethz.ch.

Table SM1: **Overview of feature sets used for the machine learning models.** EDSS: Expanded Disability Status Scale. ARAT: Action Research Arm Test. BBT: Box and Block Test. NHPT: Nine Hole Peg Test. GF: grip force. SPARC: spectral arc length. num: number. vel: velocity. TP: transport. RT: return. PA: peg approach. HA: hole approach.

Feature sets	Features	Range of values
1: patient master data	MS type	0: relapse remitting
		1: primary progressive
		2: secondary progressive
	Chronicity	25 - 76 yrs
	Sex	0: male, 1: female
2: intervention group	Intervention group	1: task-oriented high intensity
		2: task-oriented low intensity
		3: control
3: disability	EDSS	4-8
	Disability group	1: severe upper limb disability
		2: moderate upper limb disability
		3: mild upper limb disability
4: conventional scales: impairments	Motricity index	50 - 100
	Static fatigue index	13.2 - 71.3
	Monofilament index	2 - 10
	Symbol digit modality text	26 - 59
	Fahn's tremor rating scale	0 - 3
5: digital health metrics: impairments	Log jerk TP	-3.3% - 58.1%
	Log jerk RT	-38.3% - 65.7%
	SPARC RT	-38.3% - 65.7%
	Path length ratio TP	-3.0% - 54.9%
	Path length ratio RT	-7.5% - 58.9%
	Velocity max. RT	-89.1% - 90.5%
	Jerk peg approach	-3.4% - 27.9%
	GF num. peaks TP	-11.8% - 37.1%
	GF rate SPARC TP	-25.4% - 79.5%
	GF rate SPARC HA	-8.4% - 69.6%
6: conventional scales: activity	ARAT	29 - 57
	BBT	20 - 64 1/min
	NHPT	20.4 - 216.4 s

Table SM2: **Predicting intervention outcomes using data collected pre-intervention and a linear regression model.** Multiple machine learning models were trained using different feature sets (independent variables, 1-6). The training label indicated whether a considerable change across intervention was observed in a specific conventional score (dependent variable; ARAT, BBT, or NHPT). The models were evaluated in a leave-one-out cross-validation and specifically tested for one individual with strong activity limitations who did not show improvements across neurorehabilitation (referred to as unexpected non-responder). Feature set nomenclature: 1: patient master data (ms type, chronicity, age, sex). 2: intervention group. 3: disability (EDSS, disability group). 4: Conventional scales of body functions (motricity index, static fatigue index, monofilament index, symbol digit modality test, Fahn's tremor rating scale). 5: Digital health metrics of sensorimotor impairments (ten VPIT metrics). 6: Conv. scale of activity (ARAT, NHPT, BBT). The best performing (accuracy and unexpected non-responder) models relying on the least amount of features are highlighted in bold for each conventional scale. ARAT: Action Research Arm Test. BBT: Box and Block Test. NHPT: Nine Hole Peg Test. VPIT: Virtual Peg Insertion Test.

Machine learning: linear regression models						
**Feature sets**	**All participants**			**Unexpected non-responder**		
	Outcome prediction for			Outcome prediction for		
	ARAT	BBT	NHPT	ARAT	BBT	NHPT
	Balanced accuracy (%)			Correct (yes/no)		
1	88	49	36	y	y	y
2	50	37	55	y	n	y
3	36	44	36	n	y	y
4	51	60	49	n	y	n
5	33	65	**73**	n	n	y
6	83	70	48	y	y	n
1, 2	88	**74**	45	y	y	y
1, 3	81	74	29	y	y	y
1, 4	77	38	65	y	y	n
1, 5	62	46	65	y	y	y
1, 6	**89**	66	44	y	y	n
1, 2, 3	77	61	50	n	n	y
1, 4, 6	89	44	62	y	y	n
1, 5, 6	51	39	35	n	y	n
1, 4, 5, 6	65	29	69	n	y	y
1, 2, 3, 4, 5, 6	65	23	56	n	y	y

Table SM3: **Predicting intervention outcomes using pre-intervention data and decision tree models.** Multiple machine learning models were trained using different feature sets (independent variables, 1-6). The training label indicated whether a considerable change across intervention was observed in a specific conventional score (dependent variable: ARAT, BBT, or NHPT). The models were evaluated in a leave-one-out cross-validation and specifically tested for one individual with strong activity limitations who did not show improvements across neurorehabilitation (referred to as unexpected non-responder). Feature set nomenclature: 1: patient master data (ms type, chronicity, age, sex). 2: intervention group. 3: disability (EDSS, disability group). 4: Conventional scales of body functions (motricity index, static fatigue index, monofilament index, symbol digit modality test, Fahn's tremor rating scale). 5: Digital health metrics of sensorimotor impairments (ten VPIT metrics). 6: Conv. scale of activity (ARAT, NHPT, BBT). The best performing (accuracy and unexpected non-responder) models relying on the least amount of features are highlighted in bold for each conventional scale. ARAT: Action Research Arm Test. BBT: Box and Block Test. NHPT: Nine Hole Peg Test. VPIT: Virtual Peg Insertion Test.

**Machine learning: decision tree models**						
**Feature sets**	**All participants**			**Unexpected non-responder**		
	Outcome prediction for			Outcome prediction for		
	ARAT	BBT	NHPT	ARAT	BBT	NHPT
	Balanced accuracy (%)			Correct (yes/no)		
1	**88**	**83**	43	y	y	y
2	36	15	43	y	n	y
3	43	15	48	n	y	y
4	36	39	38	n	y	n
5	38	49	**49**	n	y	y
6	85	79	68	n	y	n
1, 2	88	83	43	y	y	y
1, 3	88	83	43	y	y	y
1, 4	81	83	49	n	y	n
1, 5	68	79	49	y	y	y
1, 6	80	83	68	n	y	n
1, 2, 3	88	83	43	y	y	y
1, 4, 6	80	83	60	n	y	n
1, 5, 6	80	79	37	n	y	n
1, 4, 5, 6	80	79	37	n	y	n
1, 2, 3, 4, 5, 6	80	79	37	n	y	n

Table SM4: **Predicting intervention outcomes using data collected pre-intervention and random forest models.** Multiple machine learning models were trained using different feature sets (independent variables, 1-6). The training label indicated whether a considerable change across intervention was observed in a specific conventional score (dependent variable; ARAT, BBT, or NHPT). The models were evaluated in a leave-one-out cross-validation and specifically tested for one individual with strong activity limitations who did not show improvements across neurorehabilitation (referred to as unexpected non-responder). Feature set nomenclature: 1: patient master data (ms type, chronicity, age, sex). 2: intervention group. 3: disability (EDSS, disability group). 4: Conventional scales of body functions (motricity index, static fatigue index, monofilament index, symbol digit modality test, Fahn's tremor rating scale). 5: Digital health metrics of sensorimotor impairments (ten VPIT metrics). 6: Conv. scale of activity (ARAT, NHPT, BBT). The best performing (accuracy and unexpected non-responder) models relying on the least amount of features are highlighted in bold for each conventional scale. ARAT: Action Research Arm Test. BBT: Box and Block Test. NHPT: Nine Hole Peg Test. VPIT: Virtual Peg Insertion Test.

**Machine learning: random forest models**						
**Feature sets**	**All participants**			**Unexpected non-responder**		
	Outcome prediction for			Outcome prediction for		
	ARAT	BBT	NHPT	ARAT	BBT	NHPT
	Balanced accuracy (%)			Correct (yes/no)		
1	**71**	**83**	43	y	y	y
2	32	19	39	n	n	y
3	52	26	40	y	y	y
4	43	44	63	n	y	y
5	60	54	55	y	y	y
6	76	39	63	n	y	y
1, 2	71	83	43	y	y	y
1, 3	55	83	43	y	y	y
1, 4	60	72	63	y	y	y
1, 5	60	69	63	y	y	y
1, 6	80	79	43	n	y	n
1, 2, 3	71	74	43	y	y	y
1, 4, 6	80	83	51	n	y	n
1, 5, 6	60	63	58	n	y	y
1, 4, 5, 6	63	69	**67**	n	y	y
1, 2, 3, 4, 5, 6	63	70	50	n	y	y

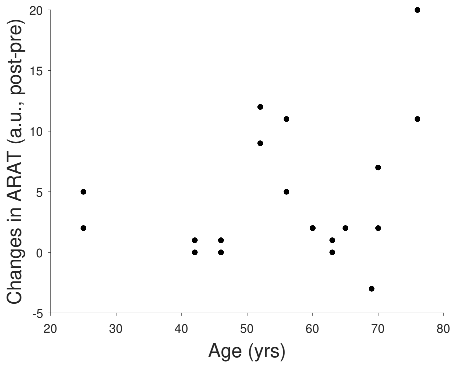

(a) r=0.29, p=0.072

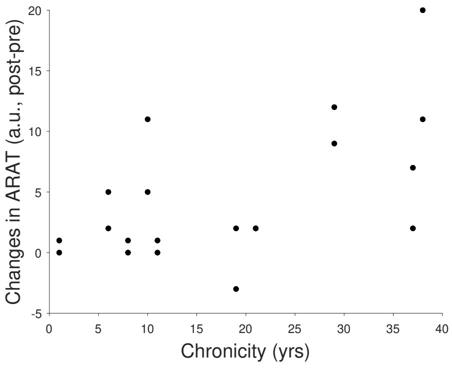

(b) r=0.58, p<0.001

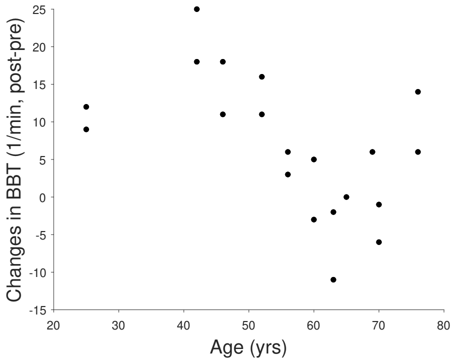

(c) r=-0.52, p<0.001

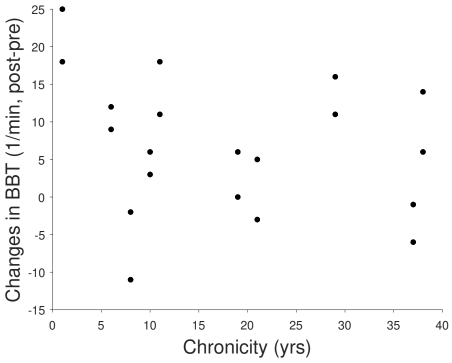

(d) r=-0.25, p=0.118

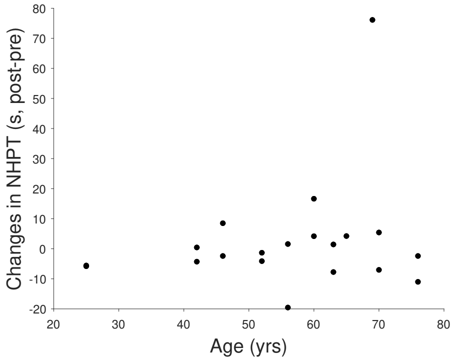

(e) r=0.22, p=0.176

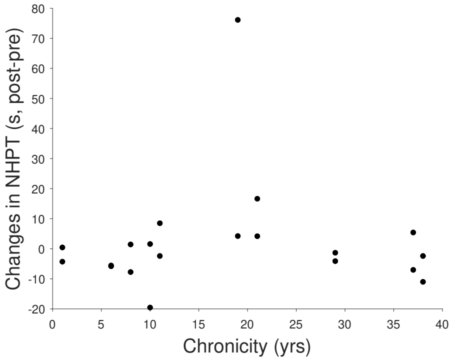

(f) r=0.03, p=0.840

Fig SM1: **Relationship between changes in clinical score (ARAT, BBT, and NHPT) and age and chronicity at baseline.** Pearson correlation analysis was performed and the correlation coefficient (r) and the corresponding p-value reported.
